# The association between pregnancy, pelvic girdle pain and health-related quality of life – a comparison of two instruments

**DOI:** 10.1186/s41687-018-0069-y

**Published:** 2018-10-01

**Authors:** Pernille Stendal Robinson, Arun Prasad Balasundaram, Nina Køpke Vøllestad, Hilde Stendal Robinson

**Affiliations:** 1 Department of Health Sciences, Institute of Health and Society, University of Oslo, P.O. Box 1089, Blindern, 0317 Oslo, Norway; 2 Present address: Department of Internal Medicine, Molde Hospital, More and Romsdal Health Trust, Molde, Norway

## Abstract

**Background:**

The main purposes in this cross-sectional study were to study the impact of pregnancy and pelvic girdle pain (PGP) on health related quality of life (HRQoL), by comparing the scores on different domains of two HRQoL instruments in pregnant women with population norms as well as in women with severe and less severe PGP. Further, to explore the association between PGP and HRQoL and whether the two instruments differ in the way they assess the influence of PGP on HRQoL.

**Methods:**

Pregnant women in gestation week 30 completed questionnaires containing the Short Form Health Survey (SF-36) and the Nottingham Health Profile (NHP). Additional variables, self-reported PGP, pain location in the pelvis and response on clinical tests were also collected. HRQoL scores were compared with expected age adjusted mean scores and comparisons between groups with different severity of PGP were made, using Mann-Whitney U, t-tests and Hodges-Lehman method.

**Results:**

Two hundred eighty-three pregnant women, mean age 31.3 (SD 4.2) years, participated. We found statistical significant differences in all domains of both HRQoL instruments in late pregnancy compared to the expected age-adjusted means of the reference populations (*p* ≤ 0.003) except for Social isolation (*p* = 0.775). Women with PGP had lower HRQoL than women without, and the most affected women scored lowest. SF-36 detected a deficit in Social Function compared to norms whereas the NHP showed no evidence of Social Isolation.

**Conclusions:**

Both instruments revealed changes in HRQoL in pregnant women compared with population norms. Pregnancy itself influences HRQoL and having PGP gave an additional impact. The consistency of the correlations between SF-36 and NHP domains across the sub-groups found in this study suggests convergent validity across levels of impairment. The results in social domains vary between SF-36 and NHP in pregnant women and might be due to the basic design (construction) of the tools.

## Background

A wide range of biochemical, physiological and structural changes occur in the female body during pregnancy [1]. Many women also experience emotional or psychological changes, and limitations in activities and participation related to their pregnancy [2]. As the measures of health-related quality of life (HRQoL) comprises physical, emotional and social dimensions of health [3], they are likely to provide relevant and adequate information regarding the impact of pregnancy.

The current literature demonstrates that HRQoL is impaired in pregnant women when using different questionnaires [2, 4–6]. Reduced HRQoL is particularly evident for domains related to physical function and some studies found that psychological and social domains appear to be unaffected by pregnancy [2, 6] while others have shown the same but also reported an increase in depressive symptoms [5].

Pelvic girdle pain (PGP) is a common complaint in pregnancy, with prevalence above 50% [2, 7–9]. PGP is defined as pain in the pelvic area, located between the posterior iliac crest and the gluteal fold, particularly near the Sacroiliac joints. The pain may radiate down posterior thigh and occur together with/or separately in the symphysis pubis. [10]. PGP is, along with back pain, the most common reason for sick leave during pregnancy in Scandinavia [11, 12], and has an impact on participation and social activities. Several studies have found that women with combined pain in the pubic symphysis (anterior part of the pelvis) and over the sacroiliac joints (posterior part of the pelvis) are more severely affected and have poorer prognosis compared with women with fewer pain sites in the pelvis [7, 13–15]. It has previously been demonstrated that women with lumbopelvic pain (combined pelvic girdle and low back pain) in late pregnancy reported lower scores on HRQoL compared to those without [2]. The differences were large for physical mobility and pain, but there was also a clear difference between the groups when considering sleep and energy. There was a less clear picture regarding social aspects, with no effect on the dimension “Social Isolation”, while the same women reported an impact of lumbopelvic pain on participation in social life. These apparently contrasting results between the dimension “Social Isolation” and impact of lumbopelvic pain on participation in social life may be due to methodological differences and, or also, validity differences in the questions asked.

Previous studies of pregnant women have used one of two HRQoL questionnaires: the Short Form Health survey (SF-36) [4, 5] or the Nottingham health profile (NHP) [2]. The basis for development of SF-36 and NHP differs. More precisely, the conceptual framework of SF-36 is based on the World Health Organization’s (WHO) definition of health [16], and includes both positive and negative statements about health. NHP, on the contrary, was developed by asking samples of patients to describe their difficulties caused by their illness. After a content analysis a set of 38 negative statements describing the consequences of ill health were chosen [17]. Hence, the NHP is based on a more negative approach compared to the SF-36 [18]. This difference is pertinent and of particular interest when assessing HRQoL in pregnant women, since pregnancy can be defined as being at the intersection between health and illness, and may influence health in both positive and negative ways.

Since the two instruments assess domains that conceptually appear to overlap (e.g. Bodily Pain and Pain, Vitality and Energy, Physical Function and Physical Mobility, Social Function and Social Isolation), results from the two instruments are often interpreted on similar general levels. Previous studies have shown strong associations between the total scores from SF-36 and NHP, as well as for domains [19–23]. However, these studies also revealed that there were low associations between some domains, such as the social ones. The studies comparing the instruments are based on specific patient groups or population samples, but we have found no studies comparing the two instruments in pregnant women.

Thus, the aims of the present study were to study the impact of pregnancy and PGP on HRQoL by: 1) Comparing the scores on different domains of SF-36 and NHP in pregnant women with population norms, and compare the scores in the women with severe and less severe pelvic girdle pain. 2) Exploring the association between PGP and HRQoL, and whether the two instruments differ in the way they assess the influence of PGP on HRQoL.

## Methods

**Fig. 1:**
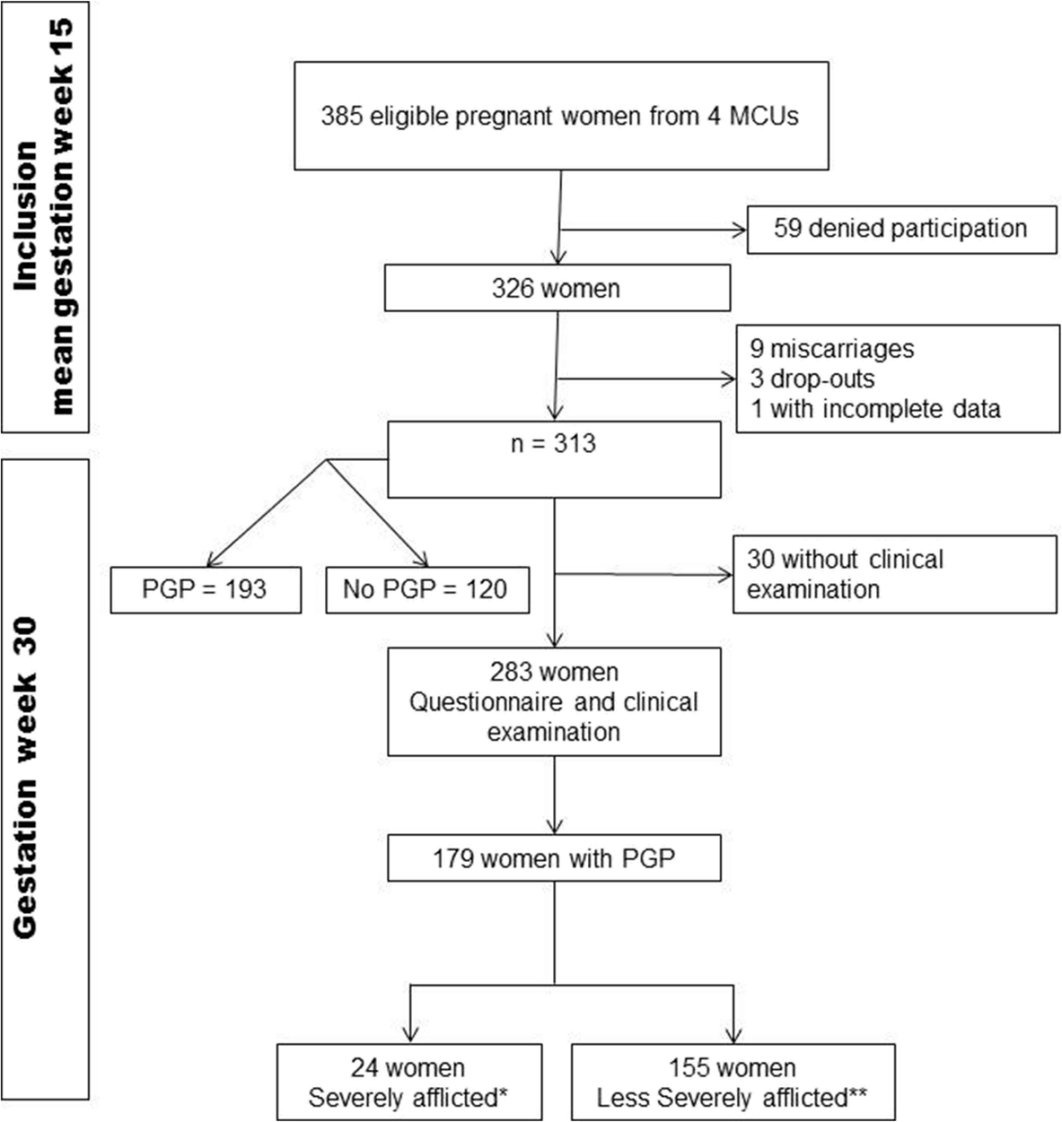
Flow chart. *Severely afflicted women (“Severe PGP”) have. 1) Combined pain in symphysis and bilateral posterior pain, AND. 2) Bilateral positive P4 test, AND. 3) ASLR score on 4 or more. **Less severely afflicted women (“Less severe PGP”) have one or two of the 1),2), 3) above

This study included cross-sectional data obtained from a previous prospective cohort study of pregnant women [9, 24]. Data were collected in collaboration with four maternity care units in and around Oslo, Norway. All Norwegian-speaking women were asked about participation at their first attendance [9, 14]. Of the 385 eligible women, 326 accepted to participate in the study. Since nine women had an early miscarriage, three dropped out and one provided incomplete data, 313 women were invited to clinical examination in gestation week (GW 30) and 283 were available (Fig. 1).

### Compliance with ethical standards

All participating women provided written informed consent. The Regional Committee for Medical and Health Research Ethics and the Norwegian Data Protection Authority approved the study (S-05284 and 13,284).

### Procedures

All participants completed standardized questionnaires including the SF-36 and the NHP in GW 15 and 30, and at 12 weeks and 1 year postpartum [24]. Furthermore, clinical examinations were conducted in GW15 and 30, and 12 weeks postpartum. In the present study, we have used only the data collected in GW 30 with the exception of demographic data collected in GW 15.

### Short form health survey (SF-36)

The SF-36 questionnaire contains 36 questions with categorical response options for 8 domains of health, including Physical Functioning, Role Physical, Role Emotional, Bodily Pain, Social Functioning, Mental Health, Vitality, and General Health perceptions [25]. The SF-36 has 8 sub-scores corresponding to the 8 domains, in which the scores are weighted sums of the questions in each domain. The scores range from 0 to 100, where lower scores indicate more disability. We used the normative values of the SF-36 available for the Norwegian population [26] to calculate expected age-adjusted means for comparisons.

### Nottingham health profile (NHP)

The NHP questionnaire contains 38 questions with a yes/no response option [17]. The instrument consists of 6 domains, including Pain, Sleep, Emotional Reactions, Social Isolation, Physical Mobility and Energy Level. The sum-scores in each dimension ranges between 0 to100, and higher scores represent more disability. The NHP is reported less sensitive to small changes in health compared to other instruments that measure similar constructs [27]. Thus, it is particularly useful in severe disease stages and also when HRQoL is severely affected. Due to a lack of Norwegian reference data, we used the normative values of the NHP available for the UK population [28] to calculate the expected age-adjusted means for comparisons. In addition, we compared our results with the results from a Swedish study on pregnant women also using the UK reference data [2].

### Self-reported pelvic girdle pain

Self-reported PGP was assessed using a single question (Do you have pain in the pelvic girdle?) with a yes/no response option. Pain intensity was addressed on a Visual analogue scale (VAS, 0–100; 100 was worst imaginable pain). Disability was measured by the Disability Rating Index (DRI), including 12 questions about difficulties performing activities of daily living, answered on visual analogue scales (VAS) [29].

### Pain drawings

We used pain-drawings [24] completed by the participants prior to the clinical examination, to determine different pain locations in the pelvic area. These were categorised as follows: no pain in the pelvic and low back area, symphysis pubis pain only, posterior pain only, combined symphysis pubis and unilateral posterior pain or combined symphysis and bilateral posterior pain. The markings on the pain-drawings were controlled with the actual areas of pain pointed out by each woman after the clinical examination. This approach was followed to maintain consistency related to the exact locations of pain in the pelvis.

### Clinical examination

The clinical examinations included the Posterior Pelvic Pain Provocation (P4) test and the functional Active Straight Leg Raise (ASLR) tests [14]. The P4 and ASLR tests have demonstrated good psychometric properties [30–32] and are recommended by the European guidelines for diagnosing PGP [10]. One of two experienced physiotherapists with postgraduate qualifications in Manual Therapy performed the clinical examinations. They were blinded for all information about the actual participant until after the examination. The tests were performed in a predefined standardized manner, responses were noted and no conclusions, concerning PGP or not, were made.

### Categorisation of severe PGP

For analysing purposes, the scores on the two clinical tests (P4 and ASLR test) and pain drawings were added to create a sum-score to categorise women into a “severe PGP” or “less severe PGP” group. “Severe PGP” was defined as the presence of all the following: bilateral positive P4 tests, ASLR sum-score of 4 or more and a combined symphysis pubis and bilateral posterior pelvic pain identified on the pain drawing. We classified the women as having “less severe PGP” if they had the presence of only one or two of the above-mentioned criteria.

### Statistical analysis

Descriptive data are presented as mean (standard deviation [*SD*]), mean (95% confidence intervals [CI]) or median (interquartile range [IQR] or 95%CI), or frequencies and percentages when appropriate. Expected age-adjusted mean scores were estimated from population norms for women using the methods proposed by Fayers and Machin (2007) [3], and accordingly a change between 5% and 10% (representing 5 to 10 points on a 100 point scale) was regarded as significant changes.

One-sample *t*-tests were used for making comparisons between the mean of the study sample and the expected age-adjusted mean scores for both instruments. For comparisons between the different groups on SF-36 scores we used t-tests and bootstrap method to calculate 95% CI’s. Mann-Whitney U tests were used for comparisons of the NHP scores between the “with PGP” and “without PGP” groups and for the “severe PGP” and “less severe PGP” groups, because these data were skewed. Hodges-Lehman’s method was used to estimate the differences in medians with 95% CI’s between the same groups. Spearman correlations were used to determine the level of association between the scores on comparable domains from the two instruments (Bodily Pain/Pain, Vitality/Energy, Physical Functioning/Physical Mobility and Social Functioning/Social Isolation). All statistical analyses were conducted using the SPSS version 24 (IBM Corp., New York, NY) with a 5% significance level.

## Results

The mean age (SD) of the whole study sample was 31.3 (4.2) years and 60% were pregnant with their first child (Table 1). The results revealed statistically significant differences in all, but one of the domains of both HRQoL instruments in late pregnancy compared to the expected age-adjusted means of the reference populations (Table 2). The only exception was for the Social Isolation domain of the NHP, which was non-significant (*p* = 0.775). Compared with the age-adjusted means from Norwegian population norms, significantly lower mean scores (worse) were found in the pregnant women for the following domains of SF-36: Physical Functioning, Role Physical, Bodily Pain, Social Functioning, Vitality and General Health Perceptions. Apart from Vitality, the mean differences were large, being between 21.7 and 48.6. Significantly higher mean scores (better) compared with population norms were also seen for Role Emotional and Mental Health, but for these domains the differences were below clinical important change. Similarly, significantly higher mean scores (worse) were found for the following domains of NHP: Energy, Sleep, Pain and Physical Mobility compared with the UK population norms, and the differences were large also for these domains (between 10.8 and 25.2).

**Table 1:** Basic characteristics of participants

	Study sample *n* = 283	Without PGP *n* = 104	With PGP *n* = 179	Less severe *n* = 155	Severe *n* = 24
	Mean (SD)				
Age (years)	31.3 (4.2)	31.6 (4.0)	31.2 (4.4)	31.1 (1.1)	31.7 (4.1)
Weight (kg)	66.5 (10.7)	64.5 (10.0)	67.7 (11.1)	67.1 (10.1)	72.0 (16)
Height (cm)	168.7 (6.1)	167.8 (6.3)	169.2 (6.0)	169.5 (5.8)	167.4 (6.8)
Body mass index	23.4 (3.5)	22.9 (3.2)	23.0 (3.7)	23.3 (3.3)	25.6 (5.1)
Education (years)	16.3 (2.6)	16.4 (2.6)	16.0 (2.6)	16.3 82.6)	16.0 (2.9)
Pain intensity	29.6 (30.7)	0 (0)	48 (26)	44 (26)	66 (22)
Disability (DRI)	37.1 (18.9)	26.4 (14.1)	43.3 (18.6)	40.6 (13.3)	60.4 (13.3)
	*n* (%)				
Married/co-habitant	275 (97)	104 (100)	171 (96)	150 (97)	21 (88)
Employed (full time)	240 (85)	91 (88)	150 (84)	132 (85)	18 (75)
Parity 0	167 (59)	70 (67)	96 (54)	87 (56)	9 (38)
1	92 (33)	27 (26)	66 (37)	53 (34)	13 (54)
> 1	24 (8)	9 (7)	17 (9)	15 (10)	2 (8)

*SD* standard deviation, *DRI* disability rating index (0–100, 100 is worst)

**Table 2:** Comparisons between the study sample and the age adjusted mean values from population norms for the SF-36 and NHP using one sample t-test

Instrument	Study sample (*n* = 283)	Age adjusted population norms^a^	*p* value	Mean difference [95%CI]
	Mean (*SD*)	Mean		
SF-36 (0–100, 100 best)				
Physical functioning	66.7 (20.5)	92.1	0.001	−25.4 [−27.5, −22.9]
Role Physical	35.4 (40.5)	84.0	0.001	−48.6 [−52.6, −43.6]
Role Emotional	86.3 (30.3)	80.8	0.003	5.5 [1.8, 8.6]
Bodily pain	55.8 (23.2)	77.3	0.001	−21.5 [−23.3, −18.2]
Social functioning	49.1 (7.2)	84.8	0.001	−35.7 [− 36.6, − 35.0]
Mental health	79.5 (11.5)	77.1	0.001	2.4 [1.4, 4.0]
Vitality	53.8 (10.7)	56.1	0.001	−2.3 [−3.4, −10]
General health perceptions	55.3 (11.1)	81.0	0.001	−25.7 [− 26.9, − 24.5]
NHP (0–100, 0 best)				
Emotional reactions	6.6 (11.5)	14.2	0.001	−7.6 [−8.9, −6.3]
Sleep	22.9 (19.6)	12.1	0.001	10.8 [8.1, 13.6]
Loss of Energy	37.5 (32.5)	15.6	0.001	22.9 [18.4, 25.7]
Pain	27.5 (27.6)	2.3	0.001	25.2 [21.3, 27.3]
Physical mobility	20.3 (19.5)	1.6	0.001	18.7 [16.3, 20.6]
Social isolation	5.9 (13.2)	6.1	0.775	−0.2 [− 1.7, 1.3]

*CI* confidence interval, *SD* standard deviation

^a^Expected age-adjusted means, computed using Norwegian (SF-36) and UK (NHP) population reference values

When comparing women “with PGP” and “without PGP”, we found significant differences in the following domains of SF-36: Physical Functioning, Role Physical, Bodily Pain, Vitality (all *p* = 0.001) and General Health Perceptions (*p* = 0.020), and in all the domains of NHP (0.001 ≤ *p* ≤ 0.016) (Table 3). Similarly, comparisons between the women with “severe PGP” and those with “less severe PGP” demonstrated significant differences only in the physical domains in both instruments, Physical Functioning, Role Physical, Bodily Pain in SF-36 (0.001 ≤ *p* ≤ 0.019), and Sleep, Energy, Pain, Physical Mobility in NHP (0.001 ≤ *p* ≤ 0.025) (Table 3). All participants without PGP or with “less severe PGP” scored 0 on the dimension Social Isolation of NHP (Tables 2 and 3). Eight of the 24 participants with severe PGP have scores above 0 (range 24.2, 43.0). A presentation of the mean differences (95% CI) for NHP data is given in Appendix 1, as an alternative presentation to Table 3.

**Table 3:** Differences between groups of women with and without self-reported PGP, and women with severe and less severe PGP on the SF-36 and NHP, using t-tests, Mann-Whitney U tests and Hodges-Lehman estimate of difference in the median of the 2 groups

Instrument	Without PGP (*n* = 104)	With PGP (*n* = 179)	*p*-value	Mean difference [95%CI]	Less severe PGP (*n* = 155)	Severe PGP (*n* = 24)	*p*-value	Mean difference [95%CI]
	Mean (SD)	Mean (SD)			Mean (SD)	Mean (SD)		
SF-36 (0–100; 100 best)								
Physical functioning	78.0 (13.0)	60.3 (21.3)	0.001	−17.7[−21.5,-14.1]	63.5 (20.1)	39.2 (16.7)	0.001	−24.3 [−31.5, − 16.7]
Role Physical	56.1 (42.6)	23.6 (34.2)	0.001	−32.5[−41.8,-23.0]	25.8 (35.2)	9.3 (23.1)	0.019	−19.5 [−26.3, −3.4]
Role Emotional	87.1 (29.2)	85.9 (31.0)	0.747	−1.2 [−8.4. 6.7]	87.1 (30.0)	77.9 (36.3)	0.110	−9.2 [−27.1, 2.8]
Bodily pain	74.3 (18.6)	44.0 (18.3)	0.001	−29.3[−34.6,-25.9]	46.8 (17.7)	33.4 (18.7)	0.001	−13.4 [−21.3,-5.3]
Social functioning	50.1 (5.6)	48.6 (7.8)	0.323	−1.5 [−3.0, 0.6]	48.1 (7.6)	52.1 (8.8)	0.049	4.0 [0.6, 8.1]
Mental health	80.9 (10.1)	78.7 (12.2)	0.080	−2.2 [−0.2, 5.3]	79.0 (11.9)	76.5 (14.1)	0.516	−2.5 [−9.3, 3.0]
Vitality	56.4 (9.9)	52.3 (10.9)	0.001	−4.1 [−6.6, −1.8]	52.5 (11.1)	50.9 (9.4)	0.378	−1.6 [−5.8, 2.5]
General health perceptions	53.4 (10.9)	56.4 (11.0)	0.020	3.0 [0.04, 5.4]	55.5 (10.5)	61.9 (13.0)	0.039	6.4 [0.5, 11.4]
	Median (IQR)	Median (IQR)	*p*-value	Intergroup comparison Median [95% CI]^a^	Median (IQR)	Median (IQR)	*p*-value	Intergroup comparison Median [95% CI]^a^
NHP (0–100; 0 best)								
Emotional reactions	0.0 (0.0, 6.5)	0.0 (0.0, 11.8)	0.016	0.0 [0.0, 0.0]	0.0 (0.0, 8.6)	8.4 (0.0, 14.3)	0.053	0.0 [0.0, 8.2]
Sleep	11.1 (0.0, 33.1)	22.0 (0.0, 22.0)	0.002	2.4 [0.0, 11.1]	22.0 (0.0, 41.7)	33.1 (22.0, 62.9)	0.025	11.1 [0.0, 22.0]
Energy	23.8 (0.0, 36.8)	23.8 (23.8, 60.6)	< 0.001	23.8 [13.0, 23.8]	23.8 (23.8, 60.6)	60.5 (23.7, 90.8)	0.006	23.8 [0.0, 36.8]
Pain	0.0 (0.0, 60.0)	38.0 (19.3, 38.3)	< 0.001	36.2 [28.2, 38.1]	37.4 (10.9, 54.7)	63.5 (41.7, 77.2)	< 0.001	26.1 [15.6, 36.5]
Physical mobility	10.1 (0.0, 10.5)	20.7 (10.1, 42.9)	< 0.001	17.5 [10.5, 20.6]	20.6 (10.1, 38.1)	45.9 (35.5, 53.2)	< 0.001	24.2 [15.1, 31.7]
Social isolation	0.0 (0.0, 0.0)	0.0 (0.0, 0.0)	0.001	0.0 [0.0, 0.0]	0.0 (0.0, 0.0)	0.0 (0.0, 24.9)	0.299	0.0 [0.0, 0.0]

*PGP* pelvic girdle pain, *SD* standard deviation, *IQR* interquartile range, ^a^ Hodges-Lehman estimate of difference in the median of the 2 groups with 95% CI

We found substantial and statistical significant associations between scores on comparable domains in the two instruments (i.e. between Vitality/Energy, Bodily Pain/Pain, Mental Health/Emotional Reactions, Physical Functioning/Physical Mobility (rho_s_ between − 0.453 and − 0.768), with two exceptions. No association was found between Social Functioning/Social Isolation (0.176 ≤ *p* ≤ 0.765) in neither of the subgroups of women. Between Vitality/Energy there was no association in the group with “severe” PGP (*p* = 0.227) (Table 4).

**Table 4:** Correlations of corresponding dimensions of SF-36 and NHP

	Participants clinically examined (*N* = 283)	Without PGP (*n* = 104)	With PGP (*n* = 179)	With less severe PGP (*n* = 155)	With severe PGP (*n* = 24)
VT/EN	−0.589**	−0.630**	−0.537**	−0.566**	−0.262
BP/P	−0.771**	− 0.519**	− 0.686**	− 0.656**	− 0.684**
MH/EM	− 0.482**	− 0.442**	− 0.495**	−0.470**	− 0.615**
SF/SI	0.049	−0.058	0.079	0.076	0.064
PF/PM	−0.742**	−0.554**	− 0.724**	−0.697**	− 0.530**

** *p* < 0.001 * 0.008 ≤ *p* < 0.002

*SF-36* Short-Form 36 (0–100, 100 is best), *NHP* Nottingham Health Profile (0–100, 0 is best), *PGP* Pelvic Girdle Pain, *VT* Vitality, *EN* Loss of Energy, *BP* Bodily Pain, *P* Pain, *MH* Mental health, *EM* Emotional reactions, *SF* Social functioning, *SI* Social isolation, *PF* Physical functioning, *PM* Physical mobility

## Discussion

The present study showed large and clinically important differences in most of the domains of HRQoL, when comparing our sample of pregnant women with the expected age-adjusted means for women from population norms. These differences were found with both HRQoL instruments. For a few domains differences were small [3] and below clinical significance. We found lower HRQoL for the pregnant women with PGP compared with those without PGP. The most affected women (“severe PGP”) showed the lowest HRQoL. These findings indicate that pregnancy influences the domains of HRQoL and that PGP is of additional importance. The consistency of the correlations between SF-36 and NHP domains across the sub-groups found in this study suggests convergent validity across levels of impairment.

Although the present study is cross-sectional, the results suggest that pregnancy has an impact on important dimensions of health. The differences to population norms were particularly large for domains related to physical function and pain. These findings were present using both instruments and are similar to the results in two previous studies [2, 4]. It is reasonable to assume that the increased loss of energy in pregnancy and reduced general health are related to the impaired physical function and increased pain.

The impact of pregnancy on HRQoL is not solely due to PGP, as the women without PGP also reported somewhat lower values than the population norms for domains related to physical function and pain. These findings indicate that pregnancy itself influences HRQoL, and that having PGP causes an additional reduction. Several factors might influence and cause reduced physical health in pregnancy irrespective of PGP and pain. Obviously, the increased weight will have an influence in itself. Furthermore, biomechanical factors such as a forward shift of gravity and altered function of the pelvis due to for instance looser ligaments [33] can have an influence. The additional influence of PGP is possibly related to pain in standing, sitting and walking and thus altered performance of weight-bearing activities [10, 14].

Furthermore, the influence of PGP appears to depend on the level of affliction, as the women with “severe PGP” had the worst scores on physical function and pain. These results are supported by a previous study, reporting that the largest differences were found between women “with” and “without” lumbopelvic pain in late pregnancy in the same NHP domains as identified in our study [2]. Furthermore, in both studies, the groups reporting pain in the lower back or pelvis scored considerable higher (worse) in these domains than the UK reference data.

In contrast to the findings on physical function, we found different results for health domains related to social life. The pregnant women reported much lower Social Functioning in SF-36 while they were comparable to population norms for the NHP scale Social Isolation. Even though social function is reduced in pregnancy, it seems not necessarily to lead to experienced social isolation. Furthermore, mainly those in the “Severe PGP” group reported Social isolation. One previous study also reported low scores on Social Isolation in late pregnancy, while the women at the same time reported limitation in participation in social activities [2]. The SF-36 focuses on how emotional and physical function affect the person’s social life (e.g. “to which extent has your physical health interfered with your normal social activities”), whereas the NHP asks how you feel (e.g. “I feel lonely”). Hence, the instruments seem to focus on and cover different degrees of severity.

The very low correlation found between the Social Functioning and Social Isolation domains also support that although scores often are compared, the domains measure different aspects of health. Hence, the two instruments supplement each other in showing that pregnancy influences on social life, without the women necessarily have a perception of this as a negative effect.

Previous studies have also reported lower associations between the social domains of the instruments in different patient populations [20, 21, 23]. Hence, there could be a reason to question the convergent validity of these domains. The two domains can easily be interpreted as measuring the same concept since they both contain the word “social”. However, our results could imply that in pregnant women, this is not the case. Pregnancy has, as previously mentioned and unlike most other conditions, not only negative impact on health. The NHP includes items such as “I feel lonely” and “I’m finding it hard to make contact with people” and the SF-36 focuses on participation in social activities. Hence, our results in pregnant women may also be quite logical: pregnancy (including PGP) may perhaps not cause a woman to find it hard to make contact, or cause her to feel lonely. However, it seems quite reasonable that she may reduce her social engagements, as found on the SF-36. The positive consequences of being pregnant, the social support during pregnancy, and the possibly lack of comorbidity, as examples, could be thought to protect against the potential negative impact of impaired physical function on the feeling of isolation. Since most of the participants in our study lived with a partner it is also possible that different findings could emerge in a study of single mothers. Furthermore, the difference in development of the instruments, is also underlying the diversity in the wording of the questions, is probably particularly evident when examining pregnant women.

This study has some noteworthy strengths and limitations. The combination of clinical examination and self- reported data is an important strength. One weakness is that different population norms were used for the two HRQoL instruments when making comparisons with the study sample. Utilising different population norms (Norwegian norms for SF-36 and UK norms for NHP) could have had an impact on the results due to the inherent cultural differences associated with it. We have tried to compensate for this by also comparing our results with the results from a Swedish study on pregnant women. However, cultural differences need to be accounted for when interpreting the results related to the HRQoL measures. The number of participants can be seen as small; however we have previously compared the women in the cohort with data from Statistics Norway and found that, when it comes to age of women giving birth and proportion of single mothers, they may be representative for pregnant women living in urban parts of Norway [34].

The skewed distribution has also created some limitations in how to compare the methods and groups. In addition, we have performed a large number of statistical tests. However, irrespective of analytical approach, we arrive at the same conclusions, and there is strength in the consistency across the main tests.

## Conclusion

Both SF-36 and NHP reveal the changes in HRQoL in pregnant women compared with expected age-adjusted population norms. The results show that pregnancy itself influences HRQoL negatively and having PGP increases this influence. We also found substantial and statistical significant associations between scores on comparable domains in the two instruments, indicating that both instruments are usable. The consistency of the correlations between SF-36 and NHP domains across the sub-groups also suggests convergent validity across levels of impairment. The results in social domains vary between SF-36 and NHP in groups of pregnant women and might be due to the differences in the basic design (construction) of the tools.

## Appendix 1

**Table 5:** Differences between groups of women with and without self-reported PGP, and women with severe and less severe PGP on the NHP, using Mann-Whitney U tests to calculate mean difference and confidence intervals from bootstrap analyses (via t-tests)

	No PGP	PGP	*p*-value	Mean difference [95%CI]	Less severe PGP	Severe PGP	*p*-value	Mean difference [95%CI]
NHP (0–100; 0 best)								
Emotional reactions	4.9 (10.3)	7.6 (12.1)	0.016	2.7 [0.4, 5.7]	7.1 (11.7)	11.0 (14.2)	0.053	3.9 [−1.7, 10.8]
Sleep	16.3 (19.5)	26.7 (26.5)	0.002	10.4 [5.9,16.0]	25.1 (26.3)	37.3 (26.3)	0.025	12.2 [2.5, 25.2]
Energy	24.9 (29.6)	44.8 (31.9)	< 0.001	19.9 [13.2,26.9]	42.5 (31.9)	60.1 (28.6)	0.006	17.6 [4.5, 31.6]
Pain	5.9 (11.3)	40.2 (26.4)	< 0.001	34.3 [29.9, 38.9]	36.7 (25.3)	62.2 (22.6)	< 0.001	25.5 [17.1, 36.9]
Physical mobility	8.9 (11.5)	27.0 (20.1)	< 0.001	18.1 [14.3, 21.7]	24.0 (18.9)	46.0 (17.3)	< 0.001	22.0 [14.6, 30.3]
Social isolation	2.7 (8.7)	7.8 (14.9)	0.001	5.1 [2.5, 8.1]	7.4 (15.0)	9.7 (14.8)	0.299	2.3 [−3.0, 9.9]

*PGP* pelvic girdle pain, *NHP* Nottingham Health profile, *CI* confidence interval

Due to skewed data we used Hodges-Lehman’s method to calculate median difference and 95%confidence interval for NHP and this is presented in the paper. This table is to show mean difference with 95%CI (also for NHP) – and will be easier to compare with expected age adjusted means for the instruments (although not appropriate due to skewness)

## Abbreviations

ASLR test: Active Straight Leg Raise test
CI: Cofidence Interval
GW: Gestation week
HRQoL: Health Related Quality of Life
NHP: Nottingham Health Profile
P4 test: Posterior Pelvic Pain Provocation test
PGP: Pelvic Girdle Pain
SD: Standard deviation
SF-36: Short Form 36
SPSS: Statistical Package for the Social Sciences
UK: United Kingdom
